# 2153. Addition of Relebactam Increases Susceptibility to Imipenem Alone against Class C β-lactamase-Positive Enterobacterales: Asia/Pacific SMART 2019-2021

**DOI:** 10.1093/ofid/ofad500.1776

**Published:** 2023-11-27

**Authors:** Sibylle Lob, Mark G Wise, Wei-Ting Chen, Fakhar Siddiqui, Katherine Young, Mary Motyl, Daniel F Sahm

**Affiliations:** Merck & Co., Inc., Schaumburg, Illinois; IHMA, Schaumburg, Illinois; MSD Taiwan, Taipei, Taipei, Taiwan; Merck & Co., Inc., Schaumburg, Illinois; Merck, Rahway, New Jersey; Merck, Rahway, New Jersey; IHMA, Schaumburg, Illinois

## Abstract

**Background:**

Imipenem/relebactam (IMI/REL) is a combination of imipenem/cilastatin with the β-lactamase inhibitor relebactam, an inhibitor of class A and C β-lactamases. We evaluated the activity of IMI/REL and comparators against AmpC- and extended-spectrum β-lactamase (ESBL)-producing *E. coli* and *K. pneumoniae* as well as against isolates of intrinsic AmpC-producing Enterobacterales species that were collected in 9 countries in Asia/Pacific as part of the global SMART surveillance program.

**Methods:**

In 2019-2021, 48 clinical laboratories in Australia, Hong Kong, Malaysia, New Zealand, Philippines, South Korea, Taiwan, Thailand, and Vietnam each collected up to 250 consecutive, aerobic or facultative, gram-negative pathogens per year from patients with bloodstream, intraabdominal, lower respiratory tract, and urinary tract infections. MICs were determined using CLSI broth microdilution and interpreted with 2023 CLSI breakpoints. Isolates that were imipenem-, IMI/REL-, or ceftolozane/tazobactam-nonsusceptible (NS) were screened for β-lactamases.

**Results:**

IMI/REL maintained activity against ≥96% of *K. pneumoniae* and *E. coli* that carried *ampC* with or without ESBL, and the addition of relebactam increased the susceptibility to imipenem alone by 13-54 percentage points among these isolates (Table). IMI/REL maintained activity against ≥92% of intrinsic *ampC* carriers, and the largest increase by addition of relebactam to imipenem alone was seen among *K. aerogenes* (25 percentage points). For imipenem-NS isolates, relebactam restored susceptibility to 98.3% of imipenem-NS *ampC*-positive *K. pneumoniae* (n=58), and to 96.8%, 97.3%, and 29.5% of imipenem-NS *Enterobacter* spp. (n=125), *K. aerogenes* (n=147), and *S. marcescens* (n=61) that carried no acquired β-lactamases, respectively.

**Figure:**
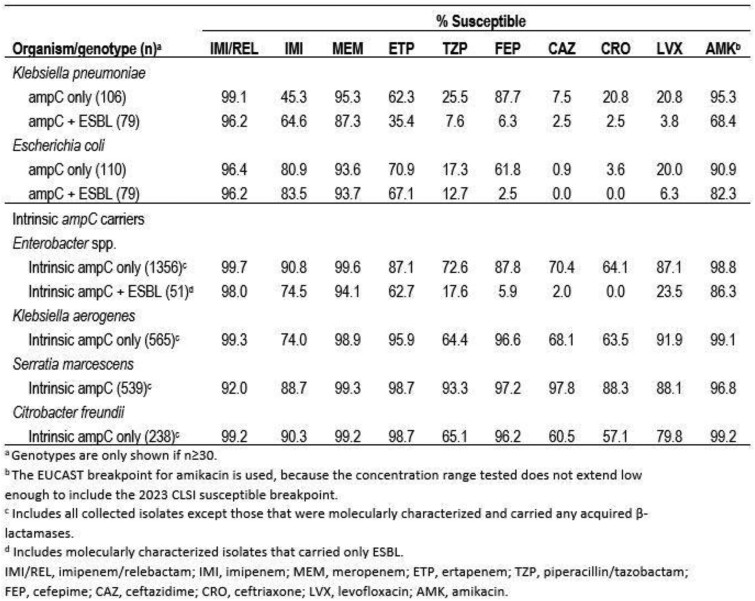

**Conclusion:**

IMI/REL showed strong activity against clinical Enterobacterales isolates that carried either acquired or intrinsic *ampC* with or without ESBL collected in Asia/Pacific. The addition of relebactam increased susceptibility to imipenem alone by up to 54 percentage points.

**Disclosures:**

**Sibylle Lob, MD**, Merck & Co., Inc.: Honoraria **Mark G Wise, PhD**, Merck & Co., Inc.: Honoraria|Pfizer Inc.: Honoraria|Venatorx: Paid fees for conducting the study and abstract preparation **Fakhar Siddiqui, MD, MBA**, Merck & Co Inc.: Employee **Daniel F. Sahm, PhD**, Merck & Co., Inc.: Honoraria|Pfizer Inc.: Honoraria|Venatorx: Paid fees for conducting the study and abstract preparation

